# Doxorubicin Is Key for the Cardiotoxicity of FAC (5-Fluorouracil + Adriamycin + Cyclophosphamide) Combination in Differentiated H9c2 Cells

**DOI:** 10.3390/biom9010021

**Published:** 2019-01-10

**Authors:** Maria Pereira-Oliveira, Ana Reis-Mendes, Félix Carvalho, Fernando Remião, Maria de Lourdes Bastos, Vera Marisa Costa

**Affiliations:** UCIBIO, REQUIMTE, Laboratory of Toxicology, Faculty of Pharmacy, University of Porto, Rua de Jorge Viterbo Ferreira, 228, 4050-313 Porto, Portugal; rosarinho_oaz@sapo.pt (M.P.-O.); afreis.mendes@gmail.com (A.R.-M.); felixdc@ff.up.pt (F.C.); remiao@ff.up.pt (F.R.); mlbastos@ff.up.pt (M.d.L.B.)

**Keywords:** doxorubicin, 5-fluorouracil, cyclophosphamide, cardiotoxicity, differentiated H9c2 cells, FAC

## Abstract

Currently, a common therapeutic approach in cancer treatment encompasses a drug combination to attain an overall better efficacy. Unfortunately, it leads to a higher incidence of severe side effects, namely cardiotoxicity. This work aimed to assess the cytotoxicity of doxorubicin (DOX, also known as Adriamycin), 5-fluorouracil (5-FU), cyclophosphamide (CYA), and their combination (5-Fluorouracil + Adriamycin + Cyclophosphamide, FAC) in H9c2 cardiac cells, for a better understanding of the contribution of each drug to FAC-induced cardiotoxicity. Differentiated H9c2 cells were exposed to pharmacological relevant concentrations of DOX (0.13–5 μM), 5-FU (0.13–5 μM), CYA (0.13–5 μM) for 24 or 48 h. Cells were also exposed to FAC mixtures (0.2, 1 or 5 μM of each drug and 50 μM 5-FU + 1 μM DOX + 50 μM CYA). DOX was the most cytotoxic drug, followed by 5-FU and lastly CYA in both cytotoxicity assays (reduction of 3-(4,5-dimethylthiazol-2-yl)-2,5-diphenyl tetrazolium bromide (MTT) and neutral red (NR) uptake). Concerning the equimolar combination with 1 or 5 μM, FAC caused similar cytotoxicity to DOX alone. Even in the presence of higher concentrations of 5-FU and CYA (50 μM 5-FU + 1 μM DOX + 50 μM CYA), 1 μM DOX was still a determinant for the cardiotoxicity observed in the cytotoxicity assays, phase contrast morphological evaluation, and mitochondrial potential depolarization evaluation. To the best of our knowledge, this was the first in vitro work with this combination regimen, DOX being the most toxic drug and key to the toxicity of FAC.

## 1. Introduction

Cancer is one of the leading causes of death worldwide. Although the number of diagnosed cases is increasing, overall cancer prognosis and 5-year survival is rising in the majority of cancer types. Early diagnosis and the extended therapeutic alternatives and strategies that exist nowadays contribute to this reality. New drugs are emerging for cancer treatments with good results, but combined chemotherapy is still the most common and well characterized option in several cancers [1,2,3]. Combined chemotherapy was, in most cases, a revolutionary step in cancer treatment, with improved prognosis or, at least, higher life time expectancy and better quality of life [4,5].

In Europe, breast cancer is the most frequently diagnosed cancer and the leading cause of cancer death among women [6]. Worldwide, it accounts for 25% of the total cancer cases (1.68 million) and 15% of the cancer deaths (520,000) [7]. Several therapies can be used, according to the characterization of the tumor or the patient. Chemotherapy is used for more than 50 years to fight breast cancer, mainly as monotherapy. However, a prospective clinical trial by the Istituto Nazionale Tumori in Milan, Italy, used a regimen called “CMF” that included an alkylating agent (cyclophosphamide (CYA)) and antimetabolites (methotrexate and 5-fluorouracil (5-FU)) [8]. That combination of agents significantly reduced the risk of breast cancer recurrence, leading to the beginning of polychemotherapy regimens that are commonly used in the clinical practice nowadays [7]. FAC is an acronym used to describe a regimen in which 5-FU, doxorubicin (DOX, also known as Adriamycin) and CYA are given by intravenous (i.v.) route, every three weeks for six cycles [7]. FAC is commonly used to treat breast cancer and was shown to be more effective than CMF [9].

Cardiotoxicity is a common side effect of anticancer drugs [3,10,11]. DOX and anthracyclines are among the drugs whose cardiotoxicity is most widely described [10]. The reports of cardiotoxicity exist since the early beginning of DOX clinical use [12] and CYA and 5-FU are also reported as cardiotoxic [3,10,11]. As expected, the use in combination of several cardiotoxic agents is conveyed to be a risk factor for the development of cardiotoxicity. Some clinical studies using FAC show cardiotoxicity between 1–6% [13,14,15], while others found values of cardiotoxicity higher than 14% in the treated patients [16,17]. In the study by Mackey, where 746 patients received FAC after which they were followed for 10 years, the conclusions were distressing [17]. Patients were subjected to 50 mg of DOX per m² followed by 500 mg of 5-FU per m², each as an i.v. infusion for 15 min, and then 500 mg of CYA per m² in an i.v. infusion for 1–5 min. Grade 3–4 heart failure occurred in 17 (2%) patients in the FAC group, and a substantial decrease in left ventricular ejection fraction (defined as a relative decrease from baseline of 20% or more) was seen in 41 (15%) patients who received this regimen [17]. Thus, DOX, CYA and 5-FU can be used together against breast cancer, but with worrying cardiotoxicity. Most authors assume that the total cumulative dose of DOX in this combined therapy is the major factor to the observed heart damage [13,14]. Nonetheless, data show higher incidence of cardiotoxicity in FAC in some studies [16,17] and the underlying mechanisms of that putative increased cardiotoxicity are overlooked. Thus, this work aimed to determine, in differentiated H9c2 cells, the cytotoxicity of DOX, 5-FU and CYA, either alone or in combination, as to ascertain the role of each drug in the cardiotoxicity of FAC. For that, equimolar mixtures of each compounds (0.2; 1 and 5 µM) were used. Nevertheless, since most reports show that the FAC regimen results in dissimilar plasma levels of the used drugs (Table 1), a mixture with higher concentrations of 5-FU and CYA than DOX was also studied to achieve more realistic and reliable results.

## 2. Materials and Methods

### 2.1. Materials

All plastic sterile material used in the cell culture was purchased to Corning-Costar (Corning, NY, USA). Phosphate-buffered saline (PBS) without calcium and magnesium and penicillin/streptomycin were obtained from Biochrom (Berlin, Germany). Fetal bovine serum (FBS), Hanks’ balanced salt solution (HBSS) and Dulbecco’s phosphate buffered saline (PBS) with calcium and magnesium were acquired to Gibco (Paisley, UK). Other compounds namely DOX hydrochloride, CYA, 5-FU, trypan blue solution (0.4%), Dulbecco’s modified eagle medium (DMEM) high glucose, sodium bicarbonate, dimethyl sulfoxide (DMSO), retinoic acid (RA), trypan blue solution (0.4%), neutral red (NR) solution, trypsin solution, 3-(4,5-dimethylthiazol-2-yl)-2,5-diphenyl tetrazolium bromide (MTT), sodium dodecyl sulfate (SDS), Hoechst 33258 solution, and paraformaldehyde were obtained from Sigma-Aldrich (Taufkirchen, Germany). 

### 2.2. Methods

#### 2.2.1. Cell Culture Experimental Protocols

The cytotoxicity of DOX, CYA, 5-FU and of their equimolar FAC mixtures (0.2; 1 or 5 µM) was assessed in differentiated H9c2 cells. Besides, a FAC mixture with concentrations based in the plasma levels of treated patients (5-FU 50 µM, DOX 1 µM, 5-CYA 50 µM) (Table 1) was also tested.

The rat cardiomyocyte derived H9c2 cell line was obtained from the European Collection of Cell Cultures (H9c2 cell line from rat (BDIX heart myoblast), from Sigma-Aldrich (Taufkirchen, Germany)). H9c2 cells were obtained from the ventricular part of a thirteenth-day rat heart embryo [18] and were maintained in a proliferative state in complete medium: DMEM with high glucose supplemented with 10% FBS and antibiotics (100 units/mL penicillin and 100 μg/mL streptomycin) at 37 °C with 5% CO_2_. All experiments were carried out before the cells reached 70–80% confluence and the cell line was used between passage 7 and 29 [19]. H9c2 cells were differentiated in a differentiation medium that comprised DMEM supplemented with 1% FBS, RA 10 nM and antibiotics. Medium was changed every other day for seven days. The differentiation was done to enhance H9c2 cardiac characteristics, as already described [19,20,21]. Cells were seeded in a density of 24,000 cells/mL [22]. All drugs studied were dissolved in PBS *per se* and, therefore, the cells were exposed to each drug. Control wells were exposed to PBS in the same volume of treated cells.

#### 2.2.2. Experimental Protocol Paradigm

After the 7-day differentiation protocol described, H9c2 cells were exposed to:

- the drugs *per se* in different concentrations (DOX (0.13–5 µM), 5-FU (0.13–5 µM), CYA (0.13–5 µM));
- equimolar mixtures of the parent drugs (FAC (0.2; 1 or 5 µM));
- FAC mixture with concentrations based in the plasma levels of treated patients (50 µM 5-FU + 1 µM DOX + 50 µM CYA) (Table 1).

Two cytotoxicity tests were done. Moreover, morphological evaluation, using phase contrast microscopy and Hoechst nuclear staining, was performed. Mitochondrial membrane potential was also assessed with the parent drugs and their combination (50 µM 5-FU + 1 µM DOX + 50 µM CYA). All these determinations will be described below.

#### 2.2.3. Cytotoxicity Tests

##### MTT Reduction Assay

The mitochondrial dehydrogenases and other reducing agents and enzymes located in other organelles reduce the water-soluble yellow tetrazolium dye, MTT, into an insoluble blue formazan that can be measured at 550 nm. The drugs *per se* were tested for 24 or 48 h, while the mixtures, FAC, were tested at 48 h. Next, the media was removed and the protocol was done according to a previously described procedure [22], in which cells were incubated for 4 h with MTT (well concentration: 0.5 mg/mL in the differentiation medium) at 37 °C. The percentage of MTT reduction of control cells was set to 100% and the values of each treatment are expressed as percentage of control cells.

##### Lysosomal Neutral Red Uptake Assay 

The amount of NR dye incorporated into the cells represents their lysosomal functionality, as this dye easily enters cell membranes and is stored in lysosomes [23]. The cells were incubated with the drugs *per se* for 24 or 48 h, whereas the effects on NR uptake assay of the mixtures, FAC, were assessed at 48 h since the majority of the cardiotoxicity observed after chemotherapy is late stage. At the end of the incubation period, cells were exposed to the differentiation medium enriched with 33 μg/mL of NR for 3 h at 37 °C. Then, the cells were washed with warm HBSS with calcium and magnesium and the NR within the cells was released adding an ethanol: acetic acid solution (50%: 1% *v*/*v*) in water. After a 15-min agitation period, the absorbance was measured at 540 nm and 690 nm [22], in a multi-well plate reader (Biotech Synergy HT (Winooski, VT, USA)). Results are presented as percentage of control cells, whose mean values were set to 100%.

#### 2.2.4. Microscopic Observation of the Cells

##### Contrast Phase Microscopy 

At 48 h, the morphology of the cells was evaluated in all possible drug combinations and respective drugs *per se* by phase contrast microscopy, using a Nikon Eclipse TS100 equipped with a Nikon DS-Fi1 camera (Tokyo, Japan). 

##### Hoechst Nuclear Staining

Hoechst staining was used to assess the effect of the drugs or their mixture on nuclear morphology of differentiated H9c2 cells. After a 48-h exposure, cells were fixed with 4% paraformaldehyde solution (10 min, 4 °C) and washed 3X with PBS containing calcium and magnesium. Cells were then stained with the nuclear dye Hoechst 33258 (final concentration 5 µg/mL) for 10 min at 37 °C (protected from light). The cells were washed with PBS containing calcium and magnesium (3X) and then observed in a Nikon Eclipse TS100 equipped with a Nikon DS-Fi1 camera using a fluorescent filter (λ excitation maximum = 346 nm and λ emission maximum = 460 nm) [22]. 

#### 2.2.5. Mitochondrial Membrane Potential 

The cells were exposed for 48 h to the drugs or their combination to assess their influence on mitochondrial membrane potential. Then, JC-1 (well final concentration of 20 µM) was added to each well. One hundred µM of the protonophore, carbonyl cyanide 3-chlorophenylhydrazone (CCCP), was used as an uncoupler of mitochondrial oxidative phosphorylation. The cells were placed in a CO_2_ incubator at 37 °C for 15 min (protected from light). In sequence, the 48-well plates were centrifuged for five min at 400× *g* at room temperature and the medium was removed carefully. In each well, 250 µL warm HBSS with calcium and magnesium were added and the plate was centrifuged for additional five min at 400× *g* at room temperature to remove the excess probe, being the supernatant aspirated carefully. This washing step was repeated three times. Fluorescence was read at a λ excitation maximum = 485 nm and a λ emission maximum = 535 nm (JC-1 exists as green monomers in apoptotic or unhealthy cells) and at a λ excitation maximum = 535 nm and a λ emission maximum = 595 nm (JC-1 forms red J-aggregates in healthy cells) in a multi-well plate reader (Biotech Synergy HT (VT, USA)). 

#### 2.2.6. Statistical Analysis

Data are expressed as mean ± standard deviation (SD). The outliers were evaluated by the Robust regression and Outlier removal (ROUT) test. The D’Agostino & Pearson normality assay was used to evaluate data distribution. When data did not follow a normal distribution, statistical analysis was performed using the Kruskal–Wallis test, followed by the Dunn’s *post-hoc* assay when a significant *p* was reached. When the distribution was normal, a parametric analysis of variance (ANOVA) was performed, followed by the Tukey’s *post hoc* test. Statistical significance was reached when *p* < 0.05. All statistical analyses were performed in GraphPad Prism 7 software (San Diego, CA, USA).

## 3. Results

### 3.1. Doxorubicin Caused A Time-Dependent Mitochondrial And Lysosomal Impairment In Differentiated H9c2 Cells

**Figure 1:**
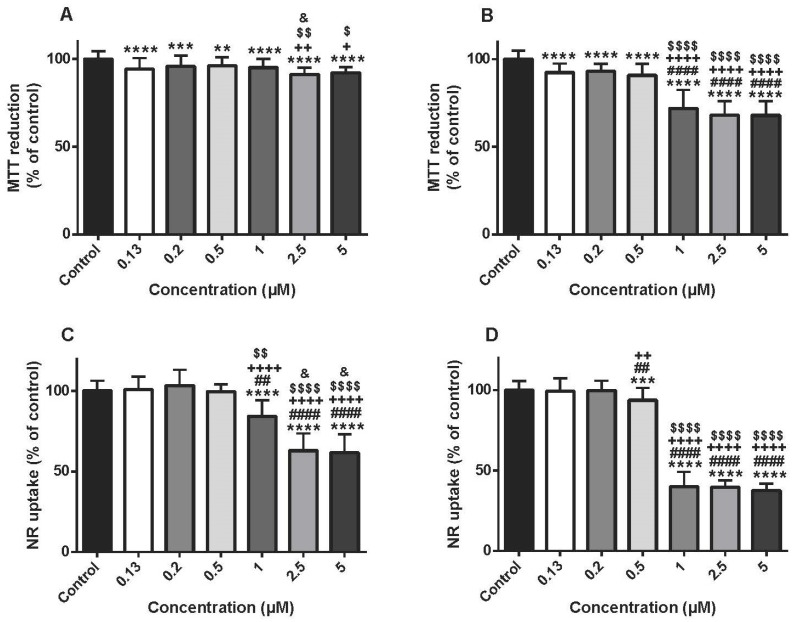
Mitochondrial and lysosomal dysfunction evaluated by the 3-(4,5-dimethylthiazol-2-yl)-2,5-diphenyl tetrazolium bromide (MTT) reduction assay (**A**,**B**) and the neutral red (NR) uptake assay (**C**,**D**), respectively, in differentiated H9c2 cells incubated with 0.13, 0.2, 0.5, 1, 2.5 and 5 μM of doxorubicin (DOX) for 24 (**A**,**C**) and 48 h (**B**,**D**). Results are presented as mean ± SD of 6–7 independent experiments (total of 30–42 wells). Statistical analyses were performed using the ANOVA test, followed by the Tukey’s *post hoc* test (**A**,**B**,**D**) and the Kruskal–Wallis test, followed by the Dunn’s *post hoc* test (**C**) (** *p* < 0.01, *** *p* < 0.001, **** *p* < 0.0001 vs. control; ^##^ *p* < 0.01, ^####^ *p* < 0.0001 vs. 0.13 µM; ^+^ *p* < 0.05, ^++^ *p* < 0.01, ^++++^ *p* < 0.0001 vs. 0.2 µM; ^$^ *p* < 0.05, ^$$^ *p* < 0.01, ^$$$$^ *p* < 0.0001 vs. 0.5 µM; ^&^ *p* < 0.05 vs. 1 µM).

The MTT reduction assay was done at two time-points (24 and 48 h) using several DOX concentrations (0.13–5 μM). Although at 24 h, DOX caused small but significant changes when compared to control cells (Figure 1A), those differences were significantly higher at 48 h, as can be seen in Figure 1B. Of note, for the three highest concentrations tested, the DOX-elicited cytotoxicity was higher when compared to the earlier time-point; however, no differences were found among them. In fact, the values of MTT reduction were, at 1 µM: 71.93 ± 10.49%, 2.5 µM: 68.10 ± 7.89% and 5 μM: 67.95 ± 8.01%, when compared to control cells (100.00 ± 4.78%) at 48 h.

The lysosomal uptake of NR was also evaluated after DOX exposure at two time-points. The three highest tested concentrations of DOX (1, 2.5 and 5 μM) caused a significant impairment of lysosomal NR uptake when compared to control cells at 24 h (Figure 1C). That cytotoxicity was further increased after a 48-h exposure, and DOX caused significant cytotoxicity at the concentrations of 5 µM (37.57 ± 4.39%), 2.5 µM (39.63 ± 4.31%), 1 µM (39.89 ± 9.33%) and 0.5 μM (93.70 ± 7.75%) when compared to control cells (100.00 ± 5.73%) (Figure 1D). 

### 3.2. The Highest Concentrations Of 5-Fluorouracil Lead To Cytotoxicity In Differentiated H9c2 Cells

**Figure 2:**
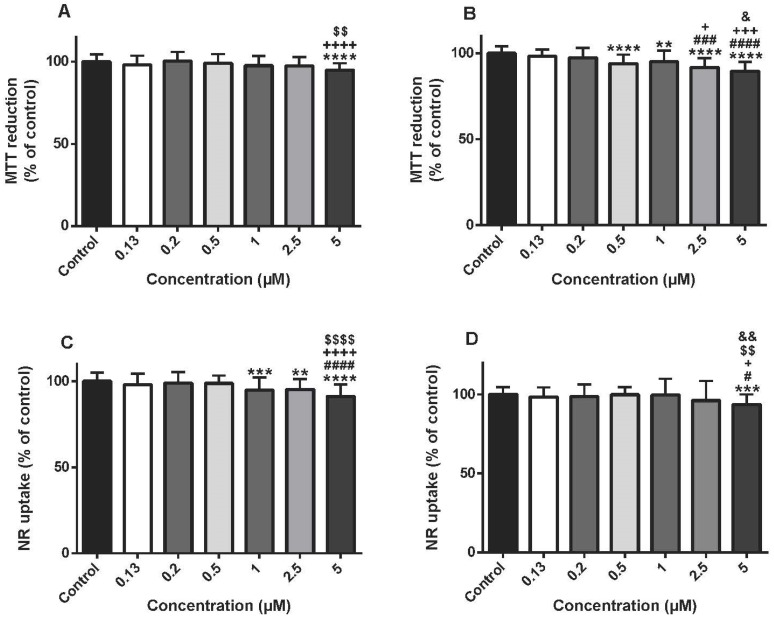
Mitochondrial and lysosomal dysfunction evaluated by the MTT reduction assay (**A**,**B**) and the NR uptake assay (**C**,**D**), respectively, in differentiated H9c2 cells incubated with 0.13, 0.2, 0.5, 1, 2.5, 5 μM of 5-fluorouracil (5-FU) for 24 (**A**,**C**) and 48 h (**B**,**D**). Results are presented as mean ± SD of 4–6 independent experiments (total of 27–54 wells). Statistical analyses were performed using the ANOVA test, followed by the Tukey’s *post hoc* test (**A**,**C**,**D**) and the Kruskal–Wallis test, followed by the Dunn’s *post hoc* test (**B**) (** *p* < 0.01, *** *p* < 0.001, **** *p* < 0.0001 vs. control; ^#^ *p* < 0.05, ^###^ *p* < 0.001, ^####^ *p* < 0.0001 vs. 0.13 µM; ^+^ *p* < 0.05, ^+++^ *p* < 0.001, ^++++^ *p* < 0.0001 vs. 0.2 µM; ^$$^ *p* < 0.01, ^$$$$^ *p* < 0.0001 vs. 0.5 µM; ^&^ *p* < 0.05, ^&&^ *p* < 0.01 vs. 1 µM).

The MTT reduction assay was assessed at two time-points (24 and 48 h) using several 5-FU concentrations (0.13–5 μM). At 24 h, no important cytotoxicity was observed for 5-FU, except for 5 µM (Figure 2A). At 48 h, 5-FU caused significant cytotoxicity at the concentrations of 5 µM (89.52 ± 5.38%), 2.5 µM (91.69 ± 5.43%), 1 µM (95.00 ± 6.51%) and 0.5 μM (93.89 ± 5.19%) when compared to control (100.00 ± 3.99%) (Figure 2B). 

The NR lysosomal uptake assay was also performed after 24-h or 48-h exposures to 5-FU (0.13–5 μM). Only at the highest concentrations of 5-FU (1, 2.5 and 5 μM) a small, but statistically, impairment of lysosomal NR uptake was found when compared to control cells at 24 h (Figure 2C). At 48 h, 5-FU caused a small but significant cytotoxicity only in the concentration of 5 µM (93.42 ± 6.62%) when compared to control cells (100.00 ± 4.63%) (Figure 2D).

### 3.3. Cyclophosphamide Was Not Cytotoxic In Differentiated H9c2 Cells In The Majority Of The Concentrations Tested

**Figure 3:**
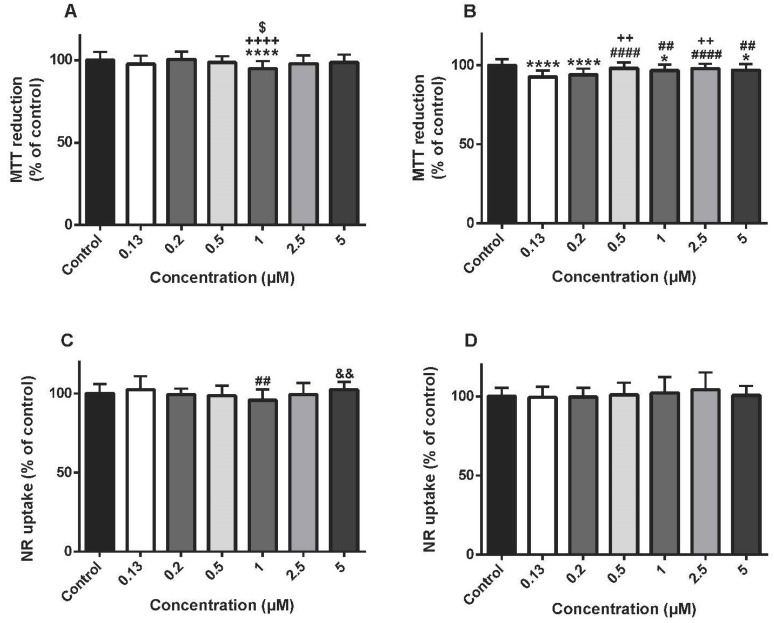
Mitochondrial and lysosomal dysfunction evaluated by the MTT reduction assay (**A**,**B**) and the NR uptake assay (**C**,**D**), respectively, in differentiated H9c2 cells incubated with 0.13, 0.2, 0.5, 1, 2.5, 5 μM of cyclophosphamide (CYA) for 24 (**A**,**C**) and 48 h (**B**,**D**). Results are presented as mean ± SD of 4–6 independent experiments (total of 20–36 wells). Statistical analyses were performed using the ANOVA test, followed by the Tukey’s *post hoc* test (**B**,**C**) and the Kruskal–Wallis test, followed by the Dunn’s *post hoc* test (**A**,**D**) (* *p* < 0.05, **** *p* < 0.0001 vs. control; ^##^ *p* < 0.01, ^####^ *p* < 0.0001 vs. 0.13 µM; ^++^ *p* < 0.01, ^++++^ *p* < 0.0001 vs. 0.2 µM; ^$^ *p* < 0.05 vs. 0.5 µM; ^&&^ *p* < 0.01 vs. 1 µM).

The CYA effects on the MTT reduction and the lysosomal NR uptake were evaluated at two time-points (24 and 48 h) using several CYA concentrations (0.13–5 μM). At 24 h, CYA 1 µM caused a significant cytotoxicity when compared to control cells, but no changes were seen in other CYA concentrations when compared to control cells (Figure 3A). At 48 h, CYA caused a small but significant cytotoxicity in the MTT reduction assay in several concentrations tested (Figure 3B). In the lysosomal NR uptake assay, no changes were seen in any concentrations or time-point evaluated when compared to control cells (Figure 3C,D). 

### 3.4. The FAC Mixture Caused Similar Cytotoxicity In The Concentration Of 1 Or 5 µM In Differentiated H9c2 Cells When Compared To Doxorubicin Per Se

**Figure 4:**
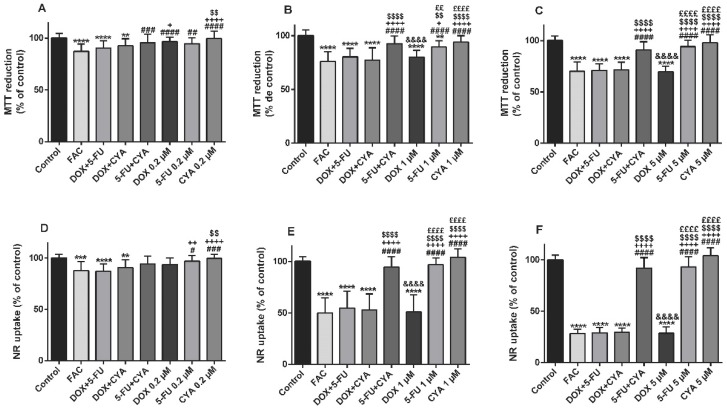
Mitochondrial and lysosomal dysfunction evaluated by the MTT reduction assay (**A**–**C**) and the NR uptake assay (**D**–**F**), respectively, in differentiated H9c2 cells incubated with 0.2 (**A**,**D**), 1 (**B**,**E**) or 5 μM (**C**,**F**) of 5-Fluorouracil + Adriamycin + Cyclophosphamide (FAC), other mixtures of DOX, 5-FU and CYA and the drugs alone for 48 h. Results are presented as mean ± SD of 4–9 independent experiments (total of 24–42 wells). Statistical analyses were performed using the ANOVA test, followed by the Tukey’s *post hoc* test (**A**) and the Kruskal–Wallis test, followed by the Dunn’s *post hoc* test (**B**–**F**) (** *p* < 0.01, *** *p* < 0.001, **** *p* < 0.0001 vs. control; ^#^ *p* < 0.05, ^##^ *p* < 0.01, ^###^ *p* < 0.001, ^####^ *p* < 0.0001 vs. FAC, ^+^ *p* < 0.05, ^++^ *p* < 0.01, ^++++^ *p* < 0.0001 vs. DOX + 5-FU; ^$$^ *p* < 0.01, ^$$$$^ *p* < 0.0001 vs. DOX+ CYA; ^&&&&^ *p* < 0.0001 vs. 5-FU+ CYA, ^££^ *p* < 0,01, ^££££^ *p* < 0.0001 vs. DOX).

FAC mixture at equimolar concentrations of 0.2, 1 or 5 μM (for each drug) was tested in differentiated H9c2 cells. In the MTT reduction assay, the FAC mixture caused cytotoxicity at the concentrations of 0.2 µM (87.14 ± 7.05%), 1 µM (76.12 ± 9.07%) and 5 μM (70.06 ± 9.33%), in a concentration-dependent manner. The mixture of DOX+5-FU and DOX+CYA also produced a significant decrease in MTT reduction ability, after a 48-h exposure to 0.2, 1 or 5 μM concentrations (Figure 4). FAC led to more cytotoxicity than 5-FU+ CYA, 5-FU or CYA, but no significant differences were found between FAC mixture and any mixture with DOX, or DOX alone at all concentrations tested, except at the 0.2 µM DOX condition. Regarding 0.2 µM DOX, the FAC mixture caused a significant higher cytotoxicity (87.14 ± 7.05%) than 0.2 µM DOX (96.55 ± 4.37%) *per se* (Figure 4A).

After a 48-h exposure to the equimolar FAC mixture at the concentration 0.2, 1 or 5 μM (each drug), the lysosomal NR uptake assay was performed. FAC mixture caused cytotoxicity at the concentrations of 0.2 µM (87.62 ± 8.95%), 1 µM (50.03 ± 14.52%) and 5 μM (28.40 ± 4.24%), in a concentration dependent manner. No significant differences were found between FAC and DOX, at any of the tested conditions (Figure 4D–F).

**Figure 5:**
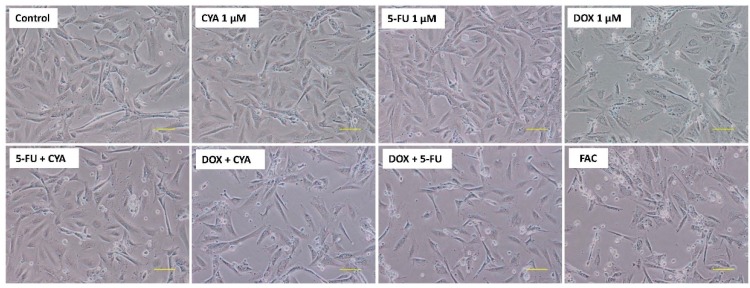
Phase contrast microscopy of differentiated H9c2 cells after a 48-h incubation with 1 μM of FAC, other mixtures of DOX, 5-FU and CYA and the drugs alone. Images are representative of two independent experiments (scale bar 100 μm).

Regarding phase contrast microscopy at 48 h, 1 µM DOX caused cellular damage, with evident cellular debris, with round and detached cells (Figure 5). There were roughly 50% less attached cells after 1 µM DOX in some fields. Similar results were found in all mixtures containing 1 µM DOX, but increased signs of cytotoxicity were observed for the FAC mixture, namely higher number of detached cells and cellular cytoplasmic damage. In fact, although cells incubated with only 1 µM CYA or 1 µM 5-FU did not show marks of cytotoxicity when compared to control cells, the mixture 5-FU+ CYA had some detached cells.

**Figure 6:**
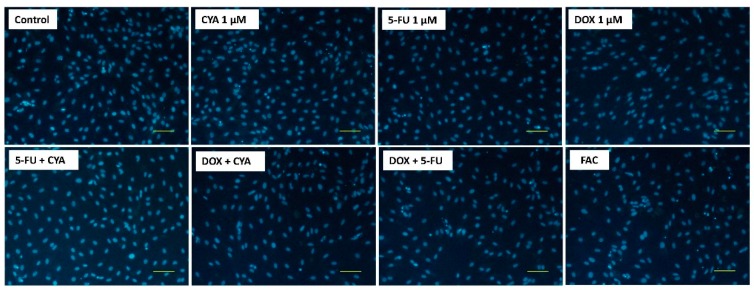
Fluorescence microscopy (Hoechst 33258 staining) of differentiated H9c2 cells after a 48-h incubation with 1 μM of FAC, other mixtures of DOX, 5-FU and CYA and the drugs alone. Images are representative of two independent experiments (scale bar 100 μm).

In the Hoechst staining, no nuclear condensation was seen in any of the conditions evaluated (Figure 6). 

### 3.5. The FAC Mixture Caused Similar Cytotoxicity To Doxorubicin Alone, When Using Concentrations Based In The Plasma Levels Found In FAC-Treated Patients

**Figure 7:**
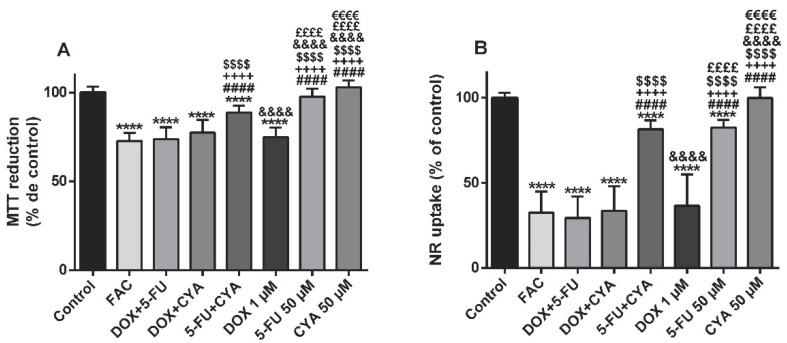
Mitochondrial and lysosomal dysfunction evaluated by the MTT reduction assay (**A**) and the NR uptake assay, (**B**) respectively, in differentiated H9c2 cells incubated with FAC, other mixtures of DOX (1 μM), 5-FU (50 μM) and CYA (50 μM) and the drugs alone for 48 h. Results are presented as mean ± SD of 4–6 independent experiments (total of 16–28 wells). Statistical analyses were performed using the ANOVA test, followed by the Tukey’s *post hoc* test (A and B) (****p < 0.0001 vs. control; ^####^ *p* < 0.0001 vs. FAC, ^++++^ *p* < 0.0001 vs. DOX + 5-FU; ^$$$$^ *p* < 0.0001 vs. DOX+ CYA; ^&&&&^ *p* < 0.0001 vs. 5-FU+ CYA, ^££££^ *p* < 0.0001 vs. DOX, ^€€€€^ *p* < 0.0001 vs. 5-FU).

The MTT reduction assay was done at 48 h to evaluate the effect of the FAC mixture at concentrations based in the plasma levels of treated patients (Table 1). This FAC mixture (50 μM 5-FU + 1 μM DOX + 50 μM CYA) caused significant cytotoxicity (72.69 ± 4.62%) when compared to control. Also DOX + 5-FU (73.65 ± 6.77%), DOX+CYA (77.44 ± 7.08%), 5-FU+CYA (88.69 ± 3.92%) and DOX alone (74.74 ± 5.52%) (Figure 7A) caused significant cytotoxicity. FAC caused a higher cytotoxicity than 5-FU+CYA, 5-FU and CYA; however, no significant differences were found between the FAC mixture and any mixture containing DOX, or DOX alone. Regarding 1 µM DOX (74.74 ± 5.52%), it caused a significant higher cytotoxicity than 50 μM 5-FU (97.69 ± 4.44%) and 50 μM CYA (102.9 ± 4.02%) (Figure 7A). The lysosomal NR uptake was also assessed at 48 h using the FAC mixture with drug concentrations based in the plasma levels of treated patients (Table 1). This FAC mixture (50 μM 5-FU + 1 μM DOX + 50 μM CYA) caused significant cytotoxicity (32.53 ± 12.37%) when compared to control cells (100.00 ± 2.82%). Nevertheless, no significant differences were found between FAC and DOX (Figure 7B). All mixtures containing two or three drugs, DOX alone or even 5-FU alone caused significant cytotoxicity when compared to control cells. Fifty μM CYA alone did not cause significant cytotoxicity in the NR uptake assay when compared to control cells. 

**Figure 8:**
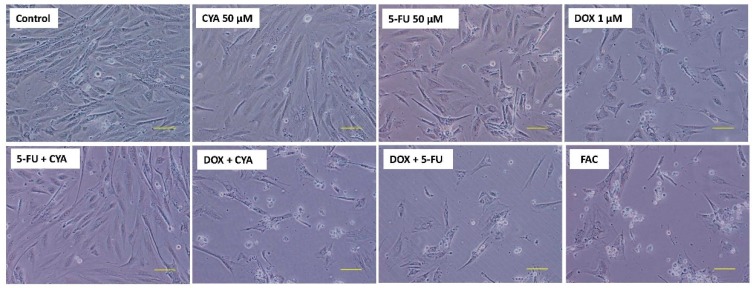
Phase contrast microscopy of differentiated H9c2 cells after 48 h incubation with FAC, other mixtures of DOX (1 μM), 5-FU (50 μM) and CYA (50 μM) and the drugs alone. Images are representative of two independent experiments (scale bar 100 μm).

**Figure 9:**
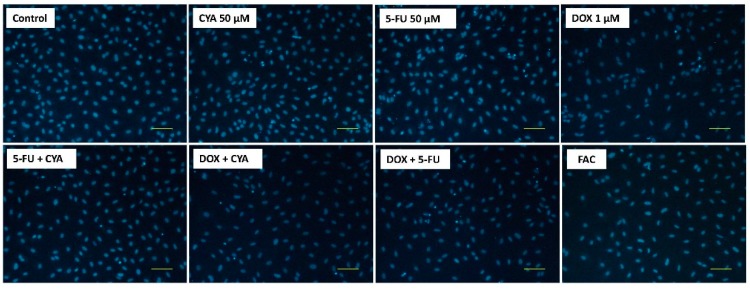
Fluorescence microscopy (Hoechst 33258 staining) of differentiated H9c2 cells after 48 h incubation with FAC, other mixtures of DOX (1 μM), 5-FU (50 μM) and CYA (50 μM) and the drugs alone. Images are representative of two independent experiments (scale bar 100 μm).

Phase contrast microscopy evaluation at 48 h of the FAC combination based in the plasma levels of treated patients showed a small increase in cytotoxicity, with higher cell detachment and the number of round cells, when compared to DOX 1 µM alone (Figure 8). Moreover, the cells exposed to only 50 μM CYA or 50 μM 5-FU elicited some cytotoxicity when compared to control cells, especially in the later condition. In the Hoescht staining, 1 µM DOX decreased the number of cells seen in the field, with very few fluorescent nuclei in the field when compared to 50 μM CYA and 50 μM 5-FU but no signs of nuclear condensation were observed (Figure 9). 

### 3.6. The FAC Mixture Caused Similar Mitochondrial Membrane Potential Depolarization To Doxorubicin Alone, When Using Concentrations Based In The Plasma Levels Found In FAC-Treated Patients

**Figure 10:**
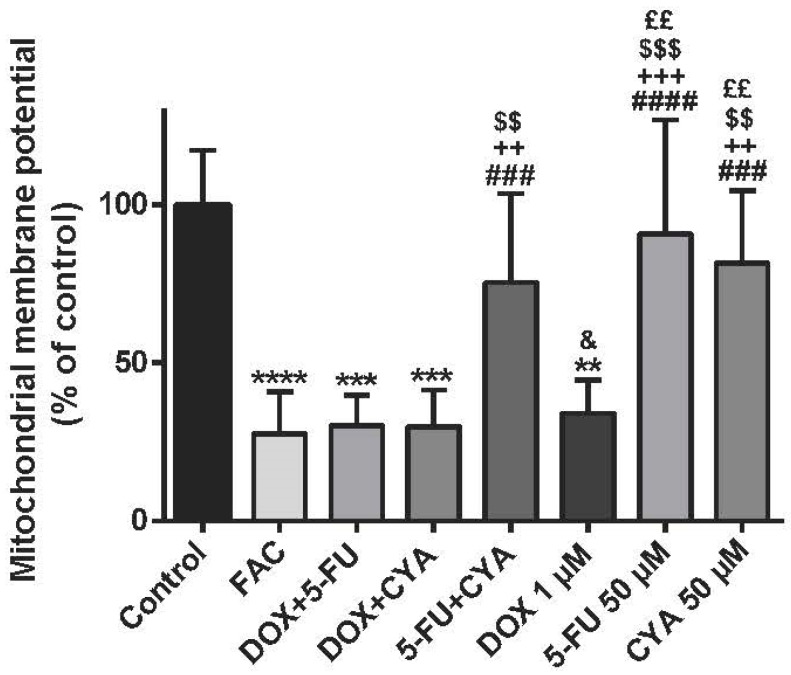
Mitochondrial membrane potential assessed by JC-1 dye in differentiated H9c2 cells incubated with combination of FAC for 48 h. Results are presented as mean ± SD of four independent experiments (total of 12 wells). Statistical analyses were performed using the Kruskal–Wallis test, followed by the Dunn’s *post hoc* test (** *p* < 0.01, *** *p* < 0.001, **** *p* < 0.0001 vs. control; ^###^ *p* < 0.001, ^####^ *p* < 0.0001 vs. FAC; ^++^ *p* < 0.01, ^+++^ *p* < 0.001 vs. DOX + 5-FU; ^$$^ *p* < 0.01, ^$$$^ *p* < 0.001 vs. DOX + CYA; ^&^ *p* < 0.05 vs. 5-FU + CYA; ^££^ *p* < 0.01 vs. DOX).

JC-1 staining showed a significant decrease in mitochondrial membrane potential of cells exposed to all mixtures containing DOX and to DOX alone when compared to the control (Figure 10). The mitochondrial membrane potential was assessed at 48 h, using the FAC combination with drug concentrations based in the plasma levels of treated patients (Table 1). FAC mixture (50 μM 5-FU + 1 μM DOX + 50 μM CYA) caused a decrease of membrane potential (27.73 ± 13.02%) when compared to control (100.00 ± 17.34%) in differentiated H9c2 cells. The mixture of DOX+5-FU (30.27 ± 9.54%), DOX+CYA (29.89 ± 11.57%) and DOX alone (34.10 ± 10.48%) also affected the mitochondrial membrane potential, after a 48-h exposure (Figure 10). FAC caused a higher decrease of mitochondrial membrane potential than 5-FU+ CYA, 5-FU and CYA; nonetheless, no significant differences were found between the FAC mixture and any mixture containing DOX, or even with DOX alone.

## 4. Discussion

This work, to the best of our knowledge, is the first to assess in vitro the cardiotoxicity of several combinations of FAC (5-FU + DOX + CYA) in differentiated H9c2 cardiac cells and to test such a large range of drug concentrations. The major findings of this work were: (i) DOX caused a time-dependent toxicity on both cytotoxicity assays performed (MTT reduction and NR uptake); (ii) 5-FU was only consistently toxic at the highest concentrations tested (5 and 50 µM), whereas CYA had the lowest toxicity in these experimental conditions; (iii) the equimolar combinations of FAC had similar toxicity when compared to DOX alone, except at the 0.2 µM mixture, while FAC caused higher toxicity than 0.2 µM DOX alone; (iv) the clinically relevant FAC mixture (50 µM 5-FU + 1 µM DOX + 50 µM CYA) had a similar toxicity when compared to 1 µM DOX, in the MTT reduction and NR uptake assays and mitochondrial membrane potential evaluation, although phase contrast microscopy showed a higher toxicity of that combination.

After the differentiation process, the cells were exposed to DOX at concentrations that ranged from 0.13 to 5 μM. According to the MTT reduction test, all tested concentrations caused toxicity to the differentiated H9c2 cells and DOX had the highest toxicity when compared to the other tested drugs. At 24 h, 5 µM DOX caused approximately a 10% decrease in MTT reduction, whereas 0.13 and 1 µM caused a decrease of about 5%, when compared to control values. In the work of Green and Leeuwenburgh, the authors tested the cytotoxicity of DOX (0.1; 1 and 10 μM) in non-differentiated H9c2 cells by the MTT reduction test after a 20 h exposure [24]. They found that 10 µM caused approximately a 30% decrease of MTT reduction, whereas 1 and 0.1 µM caused a decrease of about 10% and 5%, respectively, when compared to control cells [24]. In a study of Zhang and collaborators, non-differentiated H9c2 cells were exposed to DOX 0.2, 1 or 5 μM for 24 h. Five µM DOX caused approximately a 30% decrease in the MTT reduction, whereas 1 µM caused a decrease of about 20% when compared to control cells [25]. These data suggest that DOX is toxic regardless of the differentiation state of H9c2 cells; however, it seems that the higher H9c2 proliferation rate on non-differentiated cells contributes to amplify DOX-induced cardiotoxicity. Actually, in the lowest concentration tested in our work (0.13 µM), the cytotoxicity found in differentiated H9c2 cells was lower than a previous work that used non-differentiated H9c2 cells in similar concentrations [26]. Thus, at low concentrations, the influence of DOX in the cell cycle has a higher relevance on its cytotoxicity. On the other hand, the toxicity caused by DOX in H9c2 cells at different differentiation states (non-differentiated and differentiated with 1% FBS and with or without RA) was evaluated by the sulforhodamine B assay [27]. The authors verified that H9c2 cells differentiated with RA were more sensitive than non-differentiated cells to the effects of 0.5 μM DOX at 48 h, but no differences were seen at 1 µM [27], confirming the importance of DOX concentrations on cellular fate.

In our work, the cytotoxicity-induced by DOX was higher in the NR uptake assay than in the MTT reduction assay. To the best of our knowledge, there are no studies with DOX on differentiated H9c2 cells using the NR uptake assay. The higher sensitivity to DOX at the highest concentrations tested can be due to autophagy induction involving lysosomes [28,29]. There is evidence that the increased reactive oxygen species production induced by DOX stimulates autophagy in cardiomyocytes, acting as a mechanism to protect cells from oxidative injury [25]. Moreover, mitoxantrone has a DOX-like structure (but it is not an anthracycline) and the cytotoxicity observed in differentiated H9c2 cells after mitoxantrone exposure is also greater in the NR uptake assay than in the MTT reduction assay at similar exposure conditions [22]. 

Regarding 5-FU, only the highest concentrations caused a slight but significant cytotoxicity in differentiated H9c2 cells. Accordingly, Lamberti and collaborators showed that 5-FU is substantially less toxic in non-differentiated H9c2 cells than DOX (more than 3000× less, when comparing the half maximal inhibitory concentration, IC50) [26]. Finally, CYA was substantially less toxic in our model than the other drugs tested. Nishikawa et al. exposed non-differentiated H9c2 cells to CYA 125, 250 or 500 μM for 24 or 48 h and assessed their possible toxicity through the MTT reduction assay [30]. They observed that CYA did not lead to a significant toxicity, suggesting that CYA alone did not cause significant cytotoxicity to non-differentiated H9c2 [30]. To the best of our knowledge, there are no studies in the literature where CYA or 5-FU cytotoxicity was tested by the NR uptake assay on H9c2 cells or other cardiac models for that matter. 

As stated in the introduction section, the FAC combination led to huge increases in disease-free survival in breast cancer patients [9,14,31]. Nevertheless, it is a known fact that the concomitant use of cardiotoxic drugs increases the odds of developing cardiac problems in cancer patients [32]. In fact, the incidence of cardiotoxicity seen on FAC-treated patients can be worrying [13,14,15,16,17], although the underlying mechanisms or risk factors are largely unknown. In order to evaluate the toxicity of the FAC mixture in differentiated H9c2 cells, two paradigms were used to determine the role and to compare the toxic potency of each molecule in the mixture: (i) equimolar concentrations of 0.2; 1 and 5 μM; (ii) a combination based in the drug levels found in the plasma of treated patients (Table 1). Two cytotoxicity assays were used (the MTT reduction and the NR uptake) and cellular and nuclear morphological evaluations were undertaken. The FAC mixture, at the concentration of 0.2 μM of each drug, and after a 48-h exposure, caused significant cytotoxicity, both in the MTT reduction assay and in the NR uptake assay. The other equimolar combinations (1 and 5 µM) showed similar cytotoxicity of that seen in DOX-incubated cells (or any combination that included DOX, for that matter), whereas DOX-induced cytotoxicity was higher than the cytotoxicity of CYA, 5-FU or their combination. These results may suggest that, in the FAC regimen, DOX is the anticancer drug that contributes the most to the observed toxicity. Indeed, only the combination 1 µM 5-FU + 1 µM CYA caused a small degree of cytotoxicity, as observed by phase contrast microscopy. The incidence of chronic DOX cardiotoxicity is usually low, with an estimated incidence of about 1.7% and the incidence of DOX cardiomyopathy is primarily related to its dose. The incidence is about 4% when the dose of DOX is 500–550 mg/m^2^, 18% when the dose is 551–600 mg/m^2^, and 36% when the dose exceeds 600 mg/m^2^ [33]. Other values have been described, being the estimated cumulative percentage of patients with DOX-related heart failure 5% at a cumulative dose of 400 mg/m^2^, 26% at 550 mg/m^2^, and 48% at 700 mg/m^2^ [32]. Other risk factors for cardiomyopathy development are therapy with other cardiotoxic antitumor drugs and mediastinal radiation therapy; however, clinicians usually see the DOX cumulative dose as the major culprit for the cardiotoxicity observed after FAC [34]. Nevertheless, some studies point for higher cardiotoxicity in FAC-treated patients than expected [16,17].

In cancer patients, DOX plasma levels may be found in the range of 0.4 µM [35,36] but at peak plasma concentrations, levels higher than 1 µM can also be found [37,38,39]. At the beginning of 5-FU infusions, its levels were found to be in the range of 1.4–6.7 µM in colorectal cancer patients receiving 5-FU-based chemotherapy [40], although higher levels have been described (Table 1). Regarding CYA, the maximum concentrations in plasma following anticancer treatments can range 37–440 µM at first dose, while at the 5^th^ dose, the levels are lower than 205 µM, possibly due to enzymatic metabolic auto induction [41]. Sixty-five female patients with early or advanced breast cancer received 60 mg/m^2^ DOX over 15 min followed by 600 mg/m^2^ CYA over 15 min and the maximum observed plasma of CYA concentrations were about 100 µM [42]. The wide range of doses used with CYA and the hepatic biotransformation of CYA varies enormously from patient to patient. More information regarding plasma concentrations found after treatment with these three drugs was gathered in Table 1. Therefore, it became important in the present work to use dissimilar concentrations of the drugs in mixture, since 5-FU and CYA seem to be at higher absolute levels than DOX in the plasma of FAC-treated patients. In order to evaluate the toxicity of the FAC using concentrations based in the plasma levels of treated patients, differentiated H9c2 cells were exposed to the combination 50 µM 5-FU + 1 µM DOX + 50 µM CYA. In both the MTT reduction and the NR uptake assays, FAC caused more cytotoxicity than 5-FU+ CYA, 5-FU and CYA; however, no significant differences were found among the FAC mixture and any mixture with DOX, or even DOX alone. These results may suggest that, in the case of this combination, DOX is once again the anticancer drug that most contributes to the toxicity of the mixture. Conversely, observing cellular morphology at 48 h, a small increase in the cytotoxicity of the FAC mixture, when compared to 1 µM DOX, was seen. Still, the results again suggest that DOX may be responsible for the toxic effects observed, since cells exposed to 50 µM 5-FU, 50 µM CYA or 50 µM 5-FU + 50 µM CYA had less cells in the field but no notorious cytotoxicity in the other assays performed. In the clinical study by Dalley et al., one of the first studies in which the combination of DOX, 5-FU and CYA was used, the authors evaluated the response and toxicity of FAC in 26 patients with metastatic breast carcinoma and found that cardiotoxicity only occurred in the one patient that received more than 450 mg/m^2^ DOX [34]. Our study seems to corroborate that DOX is the major contributor to the cardiac toxicity of the FAC mixture. 

When facing these results, we evaluated the effects in mitochondrial membrane potential of the FAC mixture (50 µM 5-FU + 1 µM DOX + 50 µM CYA). DOX is a known mitochondrial toxicant [43] and we aimed to evaluate if regarding mitochondrial membrane potential, DOX was still a determinant for FAC-induced toxicity. DOX has been described as a mitochondrial toxin, either affecting mitochondrial DNA, causing cardiac mitochondrial bioenergetics changes, metabolic remodeling, or activating the intrinsic apoptotic pathway [28,44,45]. Essentially, JC-1 staining showed a significant decrease in mitochondrial potential in differentiated H9c2 cells after exposure to all mixtures containing 1 μM DOX and also 1 μM DOX alone. DOX has been shown to cause depolarization of the mitochondrial membrane [46,47] and herein, DOX was, once again, key for FAC toxicity. In a comparative study between the FAC combination and the combination docetaxel + DOX + CYA, (each combination with six treatment cycles) the presence of 5-FU did not lead to an increase in cardiotoxicity [48]. According to Azim and their collaborators, combined chemotherapeutic regimens containing no anthracyclines, such as 75 mg/m^2^ docetaxel and 600 mg/m^2^ CYA, did not increase the risk for cardiotoxicity when compared to anthracycline-containing regimens, such as the combination of 60 mg/m^2^ DOX and 600 mg/m^2^ CYA [48]. According to the studies presented, DOX is the drug that largely contributes to the cardiotoxicity of the FAC mixture, as it happens in this work, demonstrating that our paradigm can be attractive to define the toxicity mechanisms of chemotherapeutic regimens. Nonetheless, the metabolism of these drugs may be underestimated in this cellular model. Although differentiated H9c2 cells share several features of adult rat cardiomyocytes, the major metabolic in vivo biotransformation of these three drugs occurs in the liver [11]. Moreover, although they are rat cells, H9c2 cells are easy to handle and give relevant information regarding the cardiotoxicity of anticancer drugs, being widely used [22,26,27,28,29,46,47,49]. Still, further studies need to be conducted as to determine if the metabolites of FAC can contribute to its induced cardiotoxicity and other models should be used to ascertain the role of DOX in FAC-induced cardiotoxicity. Truthfully, clinical studies show high levels of cardiotoxicity development after FAC therapy [13,14,15,16,17], although other risk factors may be present. The co-administration of additional cardiotoxic agents is considered as a risk factor for the development of cardiotoxicity after anticancer therapy. Nevertheless, only trastuzumab and paclitaxel (the later shows some inconsistences in different clinical settings and the carrier cremophor EL used in the formulations with paclitaxel may be responsible for pharmacokinetic interactions) significantly and consistently increase the anthracycline-induced cardiotoxicity in real world scenarios [32].

In conclusion, this is a pioneer work that shows in vitro that DOX is the major toxicological determinant for the cardiotoxicity of FAC mixture. According to the studies presented, DOX seems to be the drug that mostly contributes to the cardiotoxicity of the FAC mixture in clinical studies, as it happens in this work, demonstrating that this mixture paradigm and cellular model can be attractive to determine the mechanisms of toxicity of the usual chemotherapeutic regimens. 

## Figures and Tables

**Table 1:** Plasma levels observed after doxorubicin (DOX), cyclophosphamide (CYA) or 5-fluorouracil (5-FU) treatments.

Drug	Patients and Dose	Plasma Concentrations	Reference
**Cyclophosphamide (CYA)**	644 plasma samples collected over a 5-year period, from 49 B-cell non-Hodgkin’s lymphoma patients 18 years receiving CYA. CYA (250 mg/m^2^) was administered as a 15-min infusion twice daily on days 2, 3 and 4 of treatment (six doses in total) (1.5 g/m^2^ total dose).	After dose 1: 37.54–438.94 μM After dose 5: 32.67–205.68 μM	[41]
	15 patients with breast cancer with previously removal of the tumor and then treated with 4 to 6 doses of injectable racemic CYA (900 or 1000 mg/m^2^ administered at intervals of 21 days, i.v.).	75.5 μM	[50]
	Patient with early stage breast cancer received CYA 600 mg/m^2^ i.v., day 1 and docetaxel 75 mg/m^2^ i.v., day 1; treatment repeated every three weeks, for 4 cycles.	187.6 μM	[51]
**Doxorubicin (DOX)**	107 children >1 year of age, with newly diagnosed acute lymphoblastic leukemia received a 24-h infusion of DOX 40 mg/m^2^.	0.04–0.61 μM	[36]
	41 children treated for newly diagnosed acute myeloid leukemia. DOX, 75 mg/m^2^ body surface area, was administered by constant i.v. infusion over 8 h.	0.32–0.60 μM	[35]
	10 female patients [normal (*n* = 3) and overweight (*n* = 7)] with breast cancer received adjuvant therapy with CYA and DOX. DOX, 60 mg/m^2^ body surface area, was administered by i.v. infusion for 40 min.	Normal: 0.07–1.16 μM Overweight: 0.08–0.68 μM	[37]
	151 Asian breast cancer patients treated with DOX-containing chemotherapy. DOX was administered at 75 mg/m^2^ as a slow bolus and docetaxel at 75 mg/m^2^ over 1 h.	0.68–1.05 μM	[39]
**5-Fluorouracil** **(5-FU)**	10 colorectal cancer patients were treated with 5-FU bolus doses ranging from 600 to 800 mg followed by a 48-h continuous infusion of 3000 to 4800 mg of FU. Blood samples were collected in the first cycle of treatment, 2 h after the start of 5-FU infusion.	1.4–6.7 μM	[40]
	40 patients with advanced colorectal cancer were treated with 5-FU plus leucovorin (LV). 5-FU was administered weekly by 8-h continuous infusion. The initial dose of 1000 mg/m^2^ was individually increased every 3 weeks by 250 mg/m^2^ steps, potentiated by 400 mg/m^2^ LV.	15.4– 23.1 μM	[52]
	22 adults with advanced gastrointestinal tract cancers and no prior systemic chemotherapy for advanced disease received interferon alpha-2a (5 MU/m^2^ subcutaneous administration, days 1–7), leucovorin (500 mg/m^2^ i.v. over 30 min, days 2–6) and 5-FU (370 mg/m^2^ i.v. over 1 h, days 2–6).	21.53–52.28 µM	[53]
**FAC**	51 patients breast cancer treated with CYA (*n* = 51) and epirubicin (*n* = 35), with or without 5-FU. The women received between 3 and 6 cycles of either 5-FU, epirubicin (EPI) and CYA (FEC) (*n* = 32), EPI and CYA (EC) (*n* = 3), DOX and CYA (AC) (*n* = 13) or 5-FU, DOX and CYA (FAC) (*n* = 3) adjuvant chemotherapy. Dose of 5-FU and CYA were 500–600 mg/m^2^. Each of the drugs was administered as a short infusion (3–20 min).	FU: 22.3–203 μM CYA: 71.4–172 μM	[54]
	28 patients with recurrent breast cancers were treated with a combination chemotherapy consisting of 5-FU (200 mg/m^2^/day P 0 days 1–28), DOX (27 mg/m^2^ i.v. at days 1 and 8), and CYA (67 mg/m^2^/day P 0 days 1–28). On the other hand, 15 patients with recurrent breast cancer were treated with a combination chemotherapy consisting of 5-FU (200 mg/m^2^/day P 0 days 1–28), CYA (67 mg/m^2^/day P 0 days 1–28) and DOX (27 mg/m^2^ i.v. at days 1 and 8).	DOX: 0.24–1.36 μM	[55]
	68 breast cancer patients received six cycles of FAC chemotherapy containing 5-FU 500 mg/m^2^, doxorubicin 50 mg/m^2^, and CYA 500 mg/m^2^ administered on the same day, every 21 days. DOX and CYA were given as “infusion-1” over 30 min followed by 5-FU as “infusion-2” over 2 h.	CYA: 9–20.2 μM	[56]

## Abbreviations

CYA	Cyclophosphamide
DMEM	Dulbecco’s modified Eagle medium
DOX	Doxorubicin
FAC	5-Fluorouracil + Adriamycin + Cyclophosphamide
FBS	Fetal bovine serum
5-FU	5-Fluorouracil
i.v.	Intravenous
HBSS	Hanks’ balanced salt solution
MTT	3-(4,5-dimethylthiazol-2-yl)-2,5-diphenyl tetrazolium bromide
NR	Neutral red
PBS	Phosphate buffered saline
RA	Retinoic acid
SD	Standard deviation
SDS	Sodium dodecyl sulfate
